# Tuberculosis as a significant cause of uveitis-related blindness: current referral trends at a tertiary uveitis center in Indonesia

**DOI:** 10.1016/j.ijregi.2025.100705

**Published:** 2025-07-18

**Authors:** Ikhwanuliman Putera, Ulifna Alfiya Sifyana, Saskia M. Rombach, Johannes R. Vingerling, P. Martin van Hagen, Rina La Distia Nora

**Affiliations:** 1 Department of Ophthalmology, Faculty Of Medicine, University of Indonesia – Cipto Mangunkusumo Hospital, Jakarta, Indonesia; 2 Department of Ophthalmology, Erasmus University Medical Center, Rotterdam, the Netherlands; 3 Department of Internal Medicine Section Allergy & Clinical Immunology, Erasmus University Medical Center, Rotterdam, the Netherlands; 4 Laboratory Medical Immunology, Department of Immunology, Erasmus University Medical Center, Rotterdam, the Netherlands; 5 Department of Immunology, Faculty of Medicine, Chulalongkorn University, Bangkok, Thailand; 6 Department of Internal Medicine, Faculty of Medicine, University of Indonesia – Cipto Mangunkusumo Hospital, Jakarta, Indonesia

**Keywords:** Blindness, Chest X-rays, Interferon-γ release assay, Tuberculosis, Uveitis

## Abstract

**Objectives:**

Uveitis may lead to blindness if improperly treated, yet the data on blindness due to uveitis, particularly, in low-resource countries, remain limited. In high–tuberculosis (TB) burden countries such as Indonesia, active systemic TB is among the leading causes of infectious uveitis. The combined impact of blindness, uveitis, and TB presents a substantial health and socioeconomic burden. This study aimed to assess the current situation of uveitis referrals, particularly, on the proportion of uveitis with active systemic TB and the proportion of blindness in TB-related uveitis.

**Methods:**

We retrospectively analyzed 1-year data from 164 newly referred patients with uveitis at a single tertiary eye Hospital in Indonesia.

**Results:**

Active systemic TB was diagnosed in approximately one in 10 patients with uveitis (16 of 164, 9.8%). At initial presentation, blindness in the worse-seeing eye was noted in 56.1% (92 of 164) of patients. The proportion of blindness was slightly higher in patients with uveitis with active systemic TB and those with no identifiable cause but positive Quantiferon-TB Gold Plus, compared with other uveitis cases, although not statistically significant (56.3%, 61.1%, and 51.3%, respectively; *P* = 0.489). Most patients with uveitis with active systemic TB (14 of 16, 87.5%) sought medical attention due to ophthalmological symptoms.

**Conclusions:**

Active TB case finding in patients with uveitis, especially in high–TB burden settings, is obligatory to prevent severe morbidity.

## Highlights

• Active systemic tuberculosis was diagnosed in approximately one in 10 patients with uveitis.
• More than half of referred patients with uveitis had blindness in worse-seeing eye.
• Eye problems were the main reason patients with uveitis with active tuberculosis sought medical care.

## Introduction

Uveitis, an inflammation of the uveal tract, may lead to irreversible blindness due to complications, such as secondary glaucoma, secondary cataract, optic neuropathy, retinal scars, macular edema, or even phthisis, if improperly treated [1]. In total, uveitis is estimated to contribute for up to 10% of blindness in developed countries [1]. However, population-based data on blindness due to uveitis in low-resource countries such as Indonesia are lacking because the current epidemiological survey remains largely focused on avoidable causes of blindness such as cataracts [2]. Notably, the prevalence of infectious and non-infectious causes of uveitis varies between high- and low-resource countries, with infectious causes being more prevalent in the latter [1]. In Indonesia, ocular toxoplasmosis, followed by uveitis associated with active systemic tuberculosis (TB), are the two leading causes of infectious uveitis [3]. From a health economic perspective, the combination of blindness, uveitis, and TB collectively impose a substantial economic burden because of direct and indirect costs. It is estimated that the direct medical costs for uveitis alone, such as those for corticosteroids or other immunosuppressants, are 4.3 times higher for patients who are blind than those without vision loss at presentation [4]. In addition, the mean direct medical cost incurred for TB treatment for each patient is estimated to be as high as US$211, with total costs, including indirect expenses, reaching up to US$1253 [5]. Therefore, it is important to objectively assess the current situation of the triple burden of uveitis, blindness, and active TB. As an initial step, (i) we aimed to report the current proportion of uveitis presented with active systemic TB among newly referred patients with uveitis and (ii) report the proportion of blindness among patients with TB-related uveitis, including a subgroup with an undetermined cause of uveitis but positive Quantiferon-TB Gold Plus (QFT) test results in addition to those with active systemic TB.

## Materials and methods

**Figure 1:**
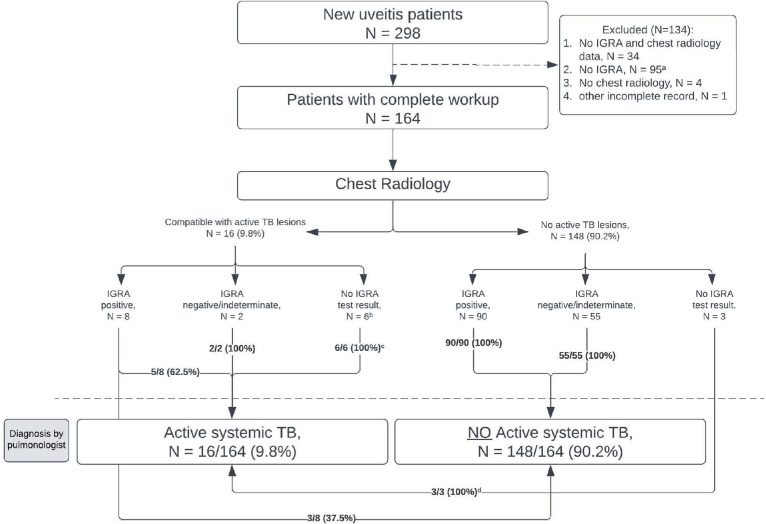
Flow of active systemic TB evaluation among patients with uveitis. Chest radiology, primarily, CXR, and the IGRA, using QFT test, were performed simultaneously during the initial workup, with a QFT cutoff of 0.35 IU/ml. Mantoux testing was far less commonly performed in our center and, therefore, was not taken into account in this analysis. ATT, anti-TB treatment; CXR, chest X-rays; IGRA, interferon-γ release assay; QFT, Quantiferon-TB Gold Plus; TB, tuberculosis. ^a^Of the 95 excluded patients without IGRA but with CXR, nine had findings suspicious for active pulmonary TB but lacked follow-up or diagnostic confirmation by pulmonologists. ^b^Four of six patients had Mantoux test performed; three (75%) showed induration ≥10 mm. ^c^Three patients had been diagnosed with active pulmonary TB and started ATT at the previous hospital, one had a history of TB, and two were diagnosed through clinical and radiological means. ^d^One patient was HIV-positive and had been on ATT for 3 months before the CXR performed at uveitis presentation, one patient had biopsy-proven TB lymphadenitis, and another, a 16-year-old patient, was diagnosed using the TB pediatric scoring system.

Between February 2023 and January 2024, 164 newly referred patients with uveitis who completed routine uveitis workup were identified at the uveitis outpatient clinic of Cipto Mangunkusumo Kirana Eye Hospital, a tertiary care hospital in Jakarta. The workup minimally included complete blood counts, liver and kidney function tests, syphilis serology, HIV screening, QFT, and chest X-ray, with additional tests to rule out other infectious and non-infectious causes of uveitis tailored based on the clinical presentation. Patients with incomplete medical record data were excluded (Figure 1). There was no significant difference in age, sex, or proportion of blindness in the worse-seeing eye between patients included and excluded from the analysis.

We retrospectively collected data on sex, age, workup results, and visual acuity at the time of initial presentation. Best-corrected visual acuity was assessed by trained nurses using a Snellen chart. For this study, blindness was defined as a best-corrected visual acuity of less than 3/60 in the worse-seeing eye because uveitis can occur unilaterally, and unilateral blindness is known to sufficiently impair quality of life [6].

Data on TB status, as assessed by pulmonologists following a national guideline for TB control [7], were retrieved. Cases without interferon-γ release assay test results were still included if the pulmonologist was able to determine the status of active disease. In addition to identifying cases of uveitis associated with active systemic TB, we categorized patients into two additional groups: those with an undetermined cause of uveitis who were QFT-positive (“QFT-positive uveitis”) and those with an established non-TB cause of uveitis or an undetermined cause with a negative QFT result (“other uveitis”).

This study was approved by the local ethical committee, Faculty of Medicine University of Indonesia (KET-850/UN2.F1/ETIK/PPM.00.02/2022).

## Results and discussion

Among the 164 patients with uveitis included in this study, 16 (9.8%) were diagnosed with active systemic TB (Figure 1). This group comprised two patients with disseminated TB (both had miliary pulmonary TB and TB lymphadenitis), one patient with active pulmonary TB with a GeneXpert test positivity from sputum sample, one with TB pleurisy, one with biopsy-proven TB lymphadenitis, and 11 patients with sputum smear–negative active pulmonary TB supported by chest radiological findings (culture was not performed). Among the latter group, one patient was also HIV-positive. Interestingly, of all 16 patients, 14 initially sought the first medical attention due to their ophthalmological symptoms.

From an ophthalmological perspective, the proportion of blindness in the worse-seeing eye among newly referred patients with uveitis was 56.1% (92 of 164 patients), whereas it was 64.4% (94 of 146 patients) in our previous data from the 2014-2015 period (Table 1). Active systemic TB cases were observed in 9.8% of patients, whereas it was 8.2% previously. In addition, the current proportion of blindness was slightly higher in patients with uveitis with active systemic TB and QFT-positive uveitis than in those with other uveitis (56.3% vs 61.1% vs 51.3%, respectively) (Table 1). The distribution of anatomical subtypes of uveitis between groups in the current cohort is presented in the Supplementary Table.

**Table 1:** Characteristics of patients with uveitis referred to our center: current situation and 2014-2015 period.

		La Distia Nora et al.’s study (2014-2015)			Current study (2023 – 2024)			
	Total patients	Active Systemic TB	QFT-positive uveitis	Other causes of uveitis	Total patients	Active Systemic TB	QFT-positive uveitis	Other causes of uveitis
Number of patients^a^	146	12/146 (8.2%)	58/146 (39.7%)	76/146 (52.1%)	164	16/164 (9.8%)	72/164 (43.9%)	76/164 (46.3%)
Characteristics								
Sex								
Female	75 (51.4%)	4 (33.3%)	42 (72.4%)	29 (38.2%)	94 (57.3%)	11 (68.8%)	45 (62.5%)	38 (50.0%)
Male	71 (48.6%)	8 (66.7%)	16 (27.6%)	47 (61.8%)	70 (42.7%)	5 (31.3%)	27 (37.5%)	38 (50.0%)
Age								
<25 years old	22 (15.1%)	2 (16.7%)	5 (8.6%)	15 (19.7%)	12 (7.3%)	4 (25.0%)	4 (5.6%)	4 (5.3%)
25-50 years old	76 (52.1%)	6 (50.0%)	28 (48.3%)	42 (55.3%)	117 (71.3%)	10 (62.5%)	53 (73.6%)	54 (71.1%)
>50 years old	48 (32.9%)	4 (33.3%)	25 (43.1%)	19 (25.0%)	35 (21.3%)	2 (12.5%)	15 (20.8%)	18 (23.7%)
Previous TB								
Yes	23 (15.8%)	0 (0%)	15 (25.9%)	8 (10.5%)	7 (4.3%)	3 (18.8%)^b^	3 (4.2%)	1 (1.3%)
No	123 (84.2%)	12 (100%)	43 (74.1%)	68 (89.5%)	157 (95.7%)	13 (81.3%)	69 (95.8%)	75 (98.7%)
Contact TB								
Yes	11 (7.5%)	1 (8.3%)	7 (12.1%)	3 (3.9%)	4 (2.4%)	0 (0%)	3 (4.2%)	1 (1.3%)
No	135 (92.5%)	11 (91.7%)	51 (87.9%)	73 (96.1%)	160 (97.6%)	16 (100%)	69 (95.8%)	75 (98.7%)
Worse-seeing eye vision category^c^								
<3/60 (blind)	94 (64.4%)	8 (66.7%)	37 (63.8%)	49 (64.5%)	92 (56.1%)	9 (56.3%)	44 (61.1%)	39 (51.3%)
≥3/60	52 (35.6%)	4 (33/3%)	21 (36.2%)	27 (35.5%)	72 (43.9%)	7 (43.8%)	28 (38.9%)	37 (48.7%)

QFT, Quantiferon-TB Gold Plus; TB, tuberculosis.

a In the 2014-2015 data, the total number of new patients was 247, of whom 146 (59.1%) were included after exclusions, including those without complete workups. In the current 2023-2024 data, 164 of 298 patients (55.0%) were included.

b The three patients with active systemic tuberculosis were tested using GeneXpert to assess rifampicin resistance, and none showed evidence of resistance. In accordance with current national guidelines (https://repository.kemkes.go.id/book/124, in Bahasa) and given the presence of active uveitis, treatment was initiated promptly in all three patients using the first line regimen 2RHZE/7RH.

c Chi-square test *P* = 0.489.

Because only four patients with active systemic TB had started antitubercular treatment (ATT) before referral to our center, we encourage that active systemic TB evaluations and confirmation be completed before referral to avoid delays in initiating ATT for those who need it. It is well-established that a single active pulmonary TB patient can infect up to 45 other individuals [8]. We speculate that patients with uveitis with active systemic (pulmonary) TB who are blind may pose a higher transmission risk because their dependence on others for daily living could increase close-contact exposure [9]. In addition, we observed that three of 16 (18.8%) cases with active systemic TB had a history of TB with ATT completion (Table 1), raising questions about whether the current active TB represents recurrence or reinfection and whether this might reflect inadequate previous ATT. Moreover, the high proportion of blindness in QFT-positive uveitis cases highlights the need to investigate whether a subset of these cases represents true extrapulmonary TB rather than merely reflecting previous *M. tuberculosis* exposure. The proportion of patients with QFT-positive uveitis in this cohort (excluding those with active systemic TB, 72 of 148 = 48.6%) exceeds the estimated prevalence of latent TB infection in the general Indonesian population, which is approximately 35% [10]. Although this study is not designed to evaluate the risks and benefits of ATT for patients with QFT-positive uveitis, this question is currently being investigated in a separate prospective study we are conducting (ClinicalTrials.gov, NCT05005637), which we anticipate will offer clearer guidance on the initiation of ATT. Recently, we reported that although several ocular features are highly suggestive of active TB uveitis, some cases may present with atypical manifestations [11]. Judicious use of advanced diagnostics, such as the polymerase chain reaction from ocular fluid we recently described [12], although not discussed here, could also be helpful after careful validation.

We acknowledge that many patients were excluded due to incomplete medical records, particularly, incomplete workups (non-completers), which may have led to over- or under-estimation of the reported proportions. Similar limitations were present in the previous study we compared with (Table 1) [3]. Some of these non-completers may arguably have had undiagnosed active TB and uveitis-related blindness due to inappropriate management. Reducing incomplete workups and maintaining patients in proper management in real-world practice is crucial to mitigating this potential public health burden.

Based on our findings, active systemic TB remains frequently observed among newly referred patients with uveitis, affecting approximately one in 10 patients with uveitis. Over half of TB-related uveitis cases present with blindness. To mitigate potential significant delays in the uveitis referral process, we strongly advocate for thorough TB evaluations in all patients with uveitis, particularly, in high–TB burden settings such as ours. ATT should be initiated promptly in patients with evidence of active systemic TB. Visual outcome after treatment and the socioeconomic impacts of this “triple burden” of blindness, uveitis, and TB require further investigations.

## Declarations of competing interest

The authors have no competing interests to declare.

## Appendix Supplementary materials

Image, application 1
