# Supplementary material for: Tuberculosis as a significant cause of uveitis-related blindness: current referral trends at a tertiary uveitis center in Indonesia
Source: IJID Reg. 2025 Jul 18;16:100705. doi: 10.1016/j.ijregi.2025.100705 (PMC12357312; doi:10.1016/j.ijregi.2025.100705)
Supplement: Supplementary file 1 [file mmc1.docx]

Supplementary Table. Anatomical subtype of uveitis of the current cohort

| Anatomical subtype of uveitis | Active Systemic TB,  N = 16, % | QFT-positive uveitis,  N = 72, % | Other causes of uveitis,  N = 76, % |
| --- | --- | --- | --- |
| Keratouveitis | 0 (0) | 3 (4.2%) | 1 (1.3%) |
| Anterior uveitis | 3 (18.8%) | 20 (27.8%) | 13 (17.1%) |
| Anterior – intermediate uveitis | 0 (0) | 2 (2.8%) | 4 (5.3%) |
| Intermediate uveitis | 0 (0) | 2 (2.8%) | 2 (2.6%) |
| Posterior uveitis | 11 (68.8%) | 14 (19.4%) | 18 (23.7%) |
| Panuveitis | 2 (12.5%) | 29 (40.3%) | 36 (47.4%) |
| Scleritis/sclera-uveitis | 0 (0) | 2 (2.8%) | 2 (2.6%) |

The anatomical subtypes of uveitis were classified according to the Standardization of Uveitis Nomenclature (SUN) criteria (Am J Ophthalmol. 2005;140(3):509–16. doi:10.1016/j.ajo.2005.03.057). Posterior uveitis includes cases with retinal vasculitis.
